# Molecular dynamics simulations of ion beam irradiation on graphene/MoS_2_ heterostructure

**DOI:** 10.1038/s41598-021-00582-2

**Published:** 2021-10-26

**Authors:** Xin Wu, Xiaobao Zhu

**Affiliations:** 1 School of Chemical Engineering and Technology, Sun Yat-Sen University, Zhuhai, 519082 Guangdong China; 2 School of Software, Nanchang Hangkong University, Nanchang, 330063 Jiangxi China

**Keywords:** Two-dimensional materials, Atomistic models

## Abstract

The interaction between ion irradiation and two-dimensional (2D) heterostructures is important for the performance modulation and application realization, while few studies have been reported. This paper investigates the influence of Ar ion irradiation on graphene/MoS_2_ heterostructure by using molecular dynamics (MD) simulations. The generation of defects is studied at first by considering the influence factors (i.e., irradiation energy, dose, stacking order, and substrate). Then uniaxial tensile test simulations are conducted to uncover the evolution of the mechanical performance of graphene/MoS_2_ heterostructure after being irradiated by ions. At last, the control rule of interlayer distance in graphene/MoS_2_ heterostructure by ion irradiation is illustrated for the actual applications. This study could provide important guidance for future application in tuning the performance of graphene/MoS_2_ heterostructure-based devices by ion beam irradiation.

## Introduction

Two-dimensional (2D) materials have become the focus of nanomaterials research since the experimental discovery of graphene in the lab in 2004^1^. Over the past two decades, more than ten thousand articles have been published every year around the topics of 2D materials. Graphene, as a single layer of graphite, is the most important 2D material. The special atomic and electronic structure has endowed graphene unparalleled properties^2,3^ and therefore, great application potentials are expected^4,5^. Besides graphene, there are also other types of 2D materials such as hexagonal boron nitride, black phosphorus, molybdenum disulphide (MoS_2_), and other transition metal dichalcogenides being discovered in the last a few years. On one hand, these 2D materials behave lots of outstanding properties similar to graphene. On the other hand, they also exhibit special performance to compensate the weakness of graphene^6^. Together with graphene, the big 2D material family opens up a new era of nanomaterials research.

During the research of 2D materials, it is found that lots of the individual 2D material has the intrinsic drawbacks which impede the full realization of the applications. For example, the high mobility of 2 × 10^5^ cm^2^ V ^−1^ s^−1^ of graphene has indicate its significant potential application in electronic devices^7^, while the graphene-based field effect transistors (FETs) generally behave low on/off switching ratios due to the zero bandgap. In contrast, 2D MoS_2_ has the direct bandgap of 1.8 eV^8^, which enables the MoS_2_-based device on/off switching ratios of > 10^8^, but the relatively low mobility limits its further applications^9^. To overcome the shortages of each material, the idea of combining different components together, i.e., generating 2D heterostructures, has emerged in recent years. 2D van der Waals (vdWs) heterostructures are one type of heterostructures which stack one layer of 2D crystal on top of another, analogous to building Lego blocks^10^. This vertical stacking feature provides the in-plane strong covalent bonds, whereas relatively weak interlayer vdW forces to the structure, which is able to integrate the merits of each 2D crystal, as well as generate some new features due to the synergistic effect, largely expanding the applications of 2D materials^11^. For example, by combining the high conductive graphene with semiconducting MoS_2_, we could fabricate high performance transparent and flexible memory devices^12^. Besides, integrating the high optical absorption and 1.8 eV direct bandgap features of MoS_2_ with high carrier mobility of graphene enables the scalable fabrication of phototransistors^13^.

The outstanding properties of 2D vdW heterostructures have promised them wide application potential similar to 2D materials. To realize the applications, a lot of efforts have been made to synthesize vdW heterostructures^14,15^. However, it should be aware that the as-synthesized vdW heterostructures generally exhibit weak interlayer coupling and uncontrolled properties, which means the postprocessing and performance modulation should be applied to the heterostructures to adjust the performance before being used. Therefore, the discovery of suitable performance modulation methods for vdW heterostructures is important. The current proposed techniques, such as mechanical-based transfer-and-stack method^16–18^, external pressure field^19^, external electric field^20^ and thermal annealing method^21^, have been demonstrated to be able to be successfully applied in tuning the interlayer coupling of vdW heterostructures. However, these methods are either too complex (e.g., transfer-and-stack method, external pressure field), or unable to be precisely controlled (e.g., thermal annealing), or relying on large external field (e.g., external pressure field). It is therefore desirable to explore new techniques to modulate the interlayer coupling in 2D vdW heterostructures for their further application.

The ion beam irradiation technique has already been demonstrated in the application of tuning the properties of 2D materials, through generating defects, or forming doping, or thinning the materials^22–24^. After ion irradiation, the mechanical, optical and electrical properties of 2D materials can be adjusted efficiently, and this process is highly dependent on the irradiation conditions (irradiation energy, irradiation dose, substrate, layer number, etc.)^25,26^. The usage of ion irradiation in tuning the performance of vdW heterostructures is less reported^27,28^, especially for graphene/MoS_2_ heterostructures, which is the most typical 2D vdW heterostructure. To uncover the performance modulating mechanism of graphene/MoS_2_ heterostructures by ion irradiation, the first thing is to make clear the ion irradiation induced phenomena in the heterostructure. In this paper, we systematically studies the influence of Ar ion irradiation on graphene/MoS_2_ heterostructure by using molecular dynamics (MD) simulations. The irradiation induced structure evolution, and the influence factors are discussed, focusing on the generated defects and the sputtered atoms. The influence of irradiation on the uniaxial tensile properties of graphene/MoS_2_ heterostructure are then investigated for the actual application under loading conditions. At last, the possibility of controlling the interlayer distance by ion irradiation are discussed. The results in this paper could provide important supports for choosing the ion irradiation method in performance modulation of 2D vdW heterostructures.

## Simulation methods

**Figure 1:**
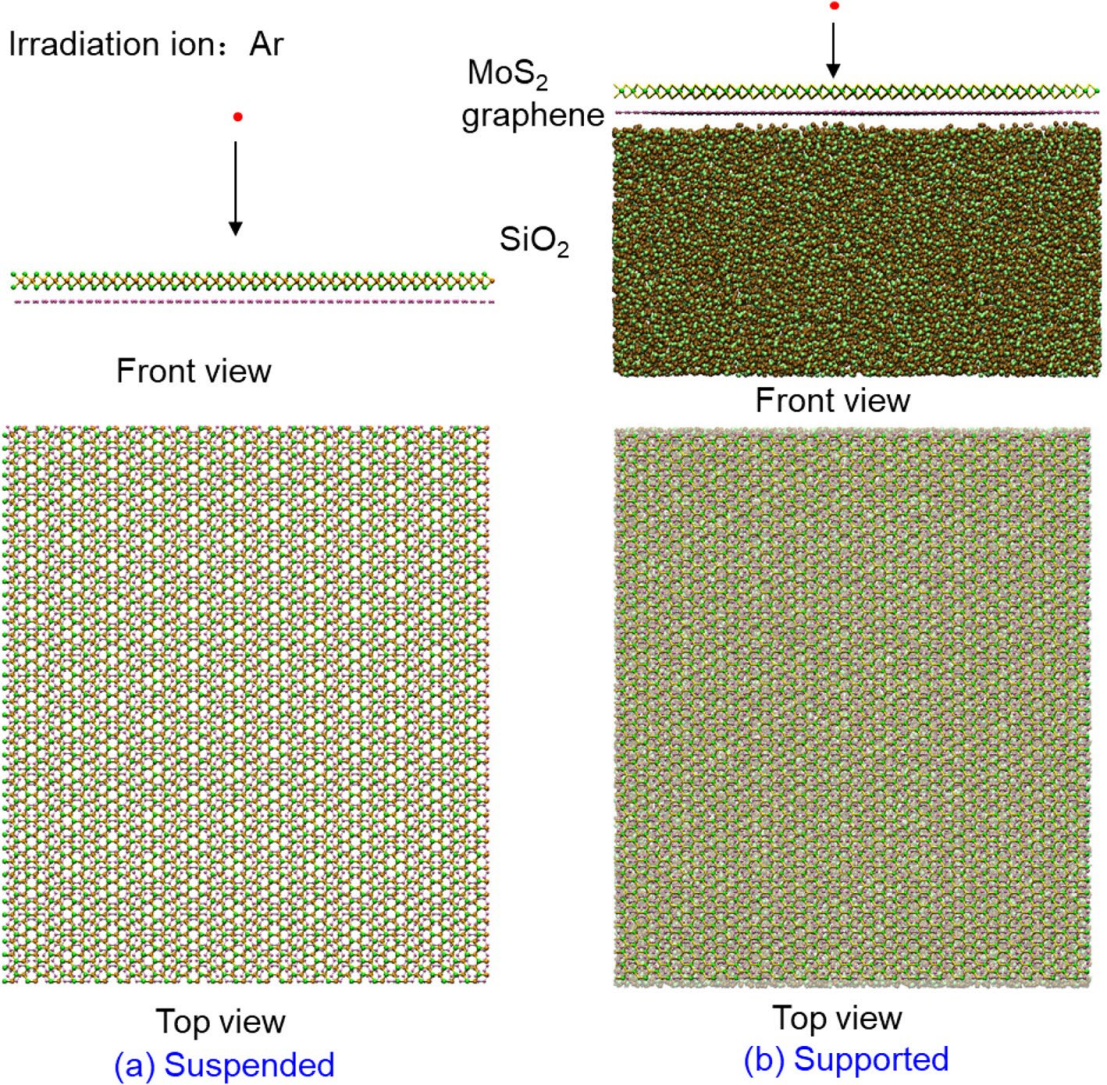
Schematic of the simulation model for (**a**) suspended and (**b**) supported cases.

For all the simulations, the large scale atomic/molecular massively parallel simulator (LAMMPS) package^29^ is adopted due to its strong capability in nanomaterials simulation. Ar ions are chosen as the representative irradiation ions, and vertically irradiated onto MoS_2_/graphene heterostructures with various energy and dose, as shown in Fig. 1. Periodic boundary conditions are applied on the structures. During the simulation, the outmost several layers of the atoms for the structure are fixed to avoid the unwanted movement, and the layers of atoms close to the fixed layers are controlled with Berendsen temperature strategy^30^ to eliminate the side effect of the pressure waves generated due to the ion irradiation. The suspended graphene/MoS_2_ heterostructure has an in-plane size of 115 × 130 Å with 11,124 atoms. The initial interlayer distance between MoS_2_ and graphene is set as 3.5 Å owing to the minimized potential energy at around this value^31^. For the supported cases, SiO_2_ is chosen as the substrate with a thickness of about 58 Å. This thickness, which results in a total number of atoms of 74,988, is enough to withstand the irradiation effect in the studied energy range. The Ar ions are randomly irradiated from a cubic box 40 Å on top of the heterostructure plane, and only the nuclei collision is considered due to much weaker energy transfer from the electron collision^32^.

To describe the multiple interatomic interactions between different atoms in the system, a hybrid potential strategy is applied. The interaction of the atoms in MoS_2_ is described by a second-generation reactive empirical bond-order (REBO) potential developed by Liang et al.^33^, which was well demonstrated by different groups^34,35^. The interactions between the carbon atoms (C–C) in graphene is captured by adaptive intermolecular reactive bond order (AIREBO) potential^36^, which could consider the formation and dissociation of covalent chemical bonds, enabling it to model the bond breaking and formation during the irradiation process. A Tersoff potential^37^ is applied to consider the interactions in SiO_2_, and the Ziegler–Biersack–Littmark (ZBL) universal repulsive potential^38^ is adopted to model the energetic collisions between Ar and the target atoms due to its strong capability in describing the interactions at small separation. The MoS_2_ and graphene layers are coupled by vdW interactions, which are considered by the Lennard–Jones (LJ) potential^39^. Besides, the interactions between graphene/MoS_2_ with the SiO_2_ substrate are also described by the LJ potential. For the parameters used in the LJ potentials (Mo-C, S-C, Mo-Si, Mo–O, S–Si, S–O, C–Si, C–O), please refer to Ref.^34,40,41^.

The MD simulation process for the ion irradiation is constituted by four stages. At the first stage, the system is relaxed at 300 K by an NVT ensemble for 5 ps to achieve an equilibrium state. At the second stage, consecutive ions with different parameters (energy and dose) are irradiated onto the heterostructure, for which the ions are emitted every 1000 timesteps and the system is kept at 300 K during the irradiation process. At the third stage, a high temperature (2000 K) annealing process is applied on the system for 30 ps to simulate the actual annealing effect in real experiment. At the last stage, the system is rescaled to room temperature (300 K) after the annealing process, and stayed at 300 K eventually. This four-stage scheme is successfully demonstrated in simulating the irradiation phenomena by different studies^23,42^. Besides the simulation of irradiation process, the mechanical properties of the irradiated structures are also revealed by uniaxial tensile simulations. The irradiated configurations are extracted from the aforementioned results and then relaxed at room temperature for enough time. Afterwards, uniaxial stretching process is applied on the structure at room temperature condition with a strain rate of 0.003 ps^-1^. It was demonstrated that the tensile strength of single-layer 2D materials would increase with strain rate^43^, while the effect maybe limited for bilayered 2D structure^44^. The value of strain rate used in this study is comparable to the previous study, and the influence of strain rate would be studied in the future research. Thereafter, the information of stress/strain is derived based on the method in Ref.^45^, then the stress–strain curve is plotted and the values of fracture stress/strain are extracted. The configurations of the stretched heterostructures are visualized by the Visual Molecular Dynamics (VMD) package^46^.

## Results and discussion

### Generation of defects

**Figure 2:**
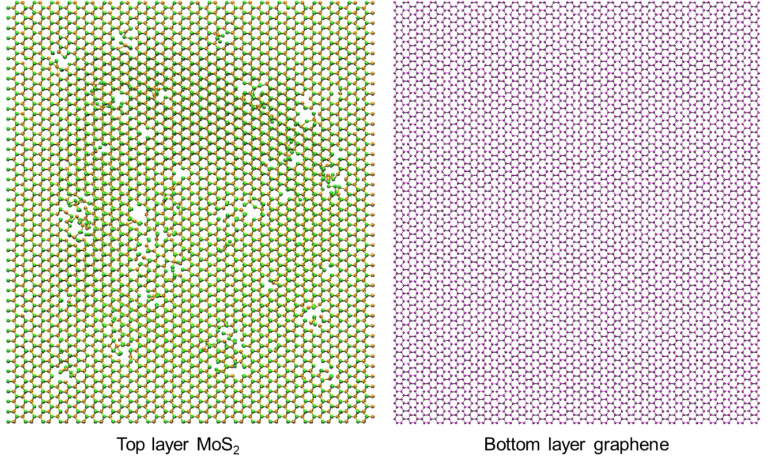
Generation of defects in suspended heterostructure layers. Irradiation energy is 100 eV and irradiation dose is 6.6 × 10^13^ /cm^2^.

Under ion irradiation, the structure of heterostructure would vibrate and the system becomes unstable. Defects would be induced into the system if the irradiation energy is high enough. As shown in Fig. 2, under ion irradiation with a parameter pair of 100 eV, 6.6 × 10^13^ /cm^2^, the top-layer MoS_2_ receives most of the impact momentum, which results in the sputtering of Mo and S atoms and generates lots of vacancy defects (monovacancies, bivacancies, and multivacancies), while the bottom-layer graphene is less influenced. Actually, under this irradiation condition, the MoS_2_ acts as a protective layer to avoid the graphene layer being damaged. The generation of vacancies in the 2D materials and 2D heterostructures can be used to modulate their properties. For example, H. K. Neupane et al.^47^ demonstrated that the vacancy defects at Mo sites could be used to efficiently tune the electronic and magnetic properties of 2D graphene/MoS_2_ heterostructure. Vacancies also affects the mechanical, electrical and magnetic properties of MoS_2_^48^. The existence of vacancies can be beneficial or detrimental to the performance of the heterostructure, depending on the location and density of the defects. Therefore, to achieve the desired properties of the heterostructure, the ability to control the defects generation is significant. The next section of this paper focuses on the dependence of the generation of vacancies on ion irradiation energy, irradiation dose, substrate, stacking order, to offer important guidance for actual applications.

### Influence of irradiation parameters and stacking order, substrate

**Figure 3:**
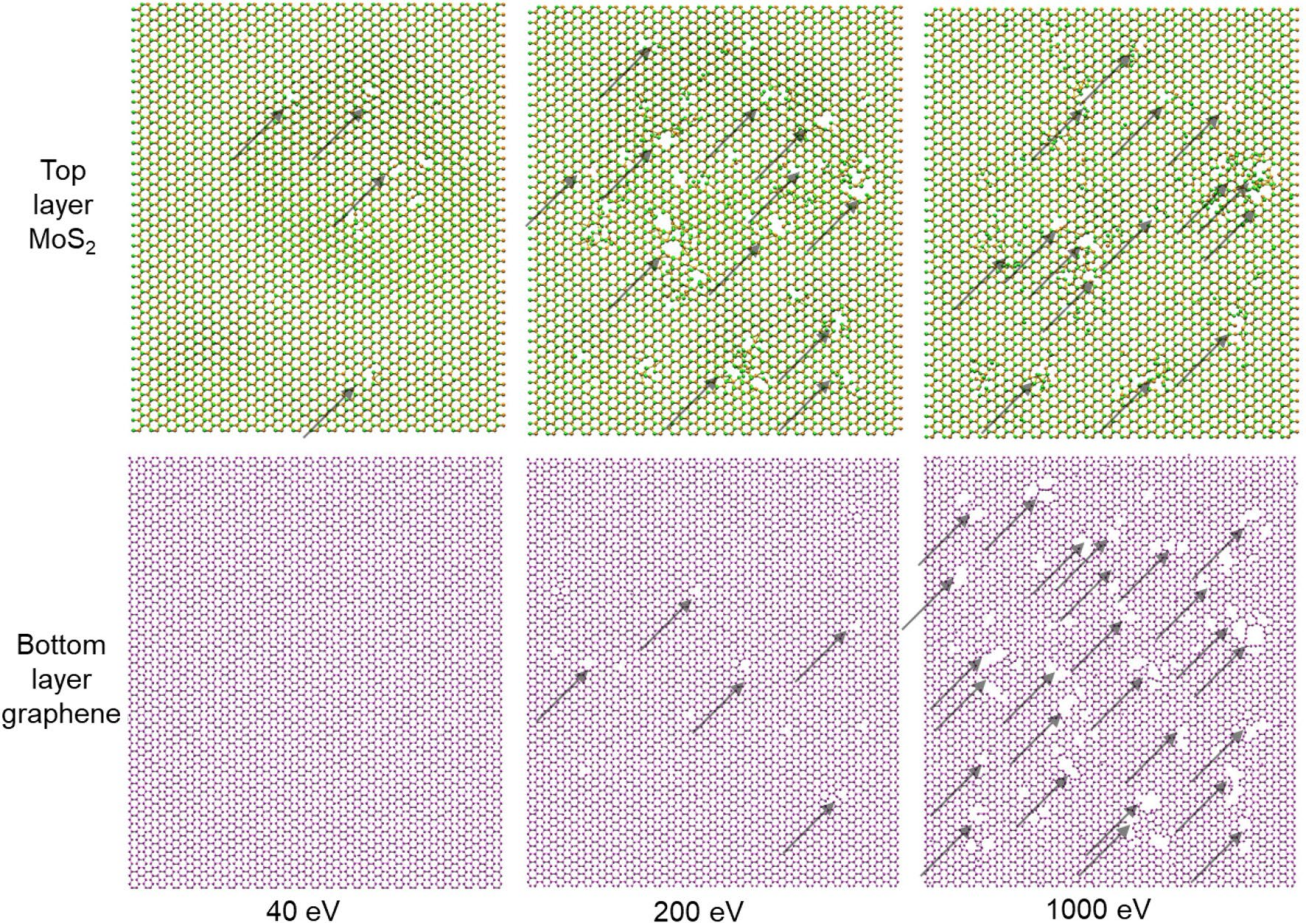
Morphologies of the suspended MoS_2_/graphene heterostructure layers under ion irradiation with different irradiation energies. The irradiation dose is 6.6 × 10^13^ /cm^2^.

For 2D materials under ion irradiation, the influence of irradiation energy and dose on the defect formation is important, so do 2D heterostructures. As shown in Fig. 3, under low irradiation energy (40 eV), the generation of defects in top-layer MoS_2_ is limited, and the bottom-layer graphene is fully protected. With the increase of irradiation energy (200 eV), the generation of vacancies in both MoS_2_ and graphene would be intensified. Under this situation, the defects in top-layer MoS_2_ would be more severe than in bottom-layer graphene. If the irradiation energy is further increased (1000 eV), the defects in MoS_2_ is slightly changed, while the defects in graphene increase a lot, generating a more severe defect formation in graphene. For the low energy irradiation case, very little energy traverse through the top-layer plane, and the bottom layer is well protected. While for the high energy irradiation case, some energetic ions pass through top-layer, and collide with the bottom-layer, inducing the direct vacancy formation in the graphene. Besides, the sputtered Mo and S atoms from the top-layer MoS_2_ are also energetic at this condition, and they would fly to the graphene and lead to the second collision and indirect vacancy formation in the graphene layer. The generation of indirect defects in graphene become dominate if the ion energy is high enough, which could explain the reversed phenomenon observed in the 1000 eV case.

**Figure 4:**
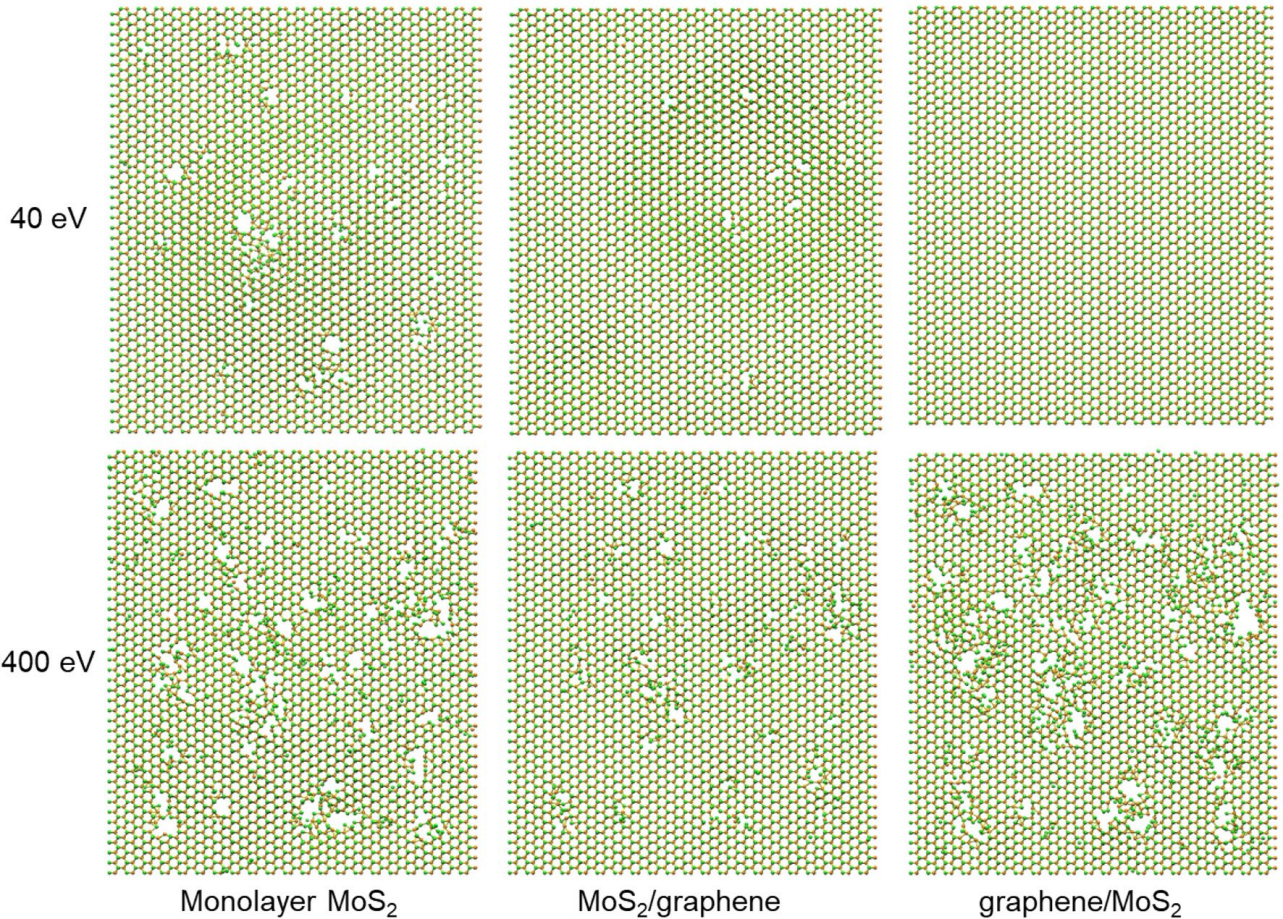
Comparison of the morphologies of MoS_2_ under irradiation with low (40 eV) and high (400 eV) energies for the study cases of monolayer MoS_2_, MoS_2_/graphene heterostructure, and graphene/MoS_2_ heterostructure.

The influence of the stacking order is investigated by switching the MoS_2_ and graphene layers, to obtain a graphene/MoS_2_ heterostructure, so that top-layer graphene acts as a protective layer to MoS_2_. Figure 4 shows the comparison of the defect formation in MoS_2_ layer for MoS_2_/graphene and graphene/MoS_2_ heterostructures. The case study of monolayer MoS_2_ is also provided as a reference. It shows that under the low ion irradiation energy (40 eV), the defects of MoS_2_ in the two types of heterostructures are always fewer than the pure monolayer MoS_2_ case, indicating a protective effect of the graphene to MoS_2_ layer, regardless of the stacking order of heterostructures. In the graphene/MoS_2_ heterostructure, the shielding effect of top-layer graphene could mostly relieve the irradiation damage onto bottom-layer MoS_2_, while in MoS_2_/graphene heterostructure, it is the interaction between bottom-layer graphene and top-layer MoS_2_ weakening the irradiation damage onto MoS_2_. If the irradiation energy is increased to 400 eV, it is found that the bottom graphene can still reduce the irradiation effect of MoS_2_. While for the graphene on top (graphene/MoS_2_ case), the irradiation damage is more severe, which is attribute to the indirect collision from the sputtered carbon atoms in top graphene layer. For the top-layer 2D crystal, the switch from protective effect to destructive effect at different energy levels corresponds to the results in Fig. 3.

**Figure 5:**
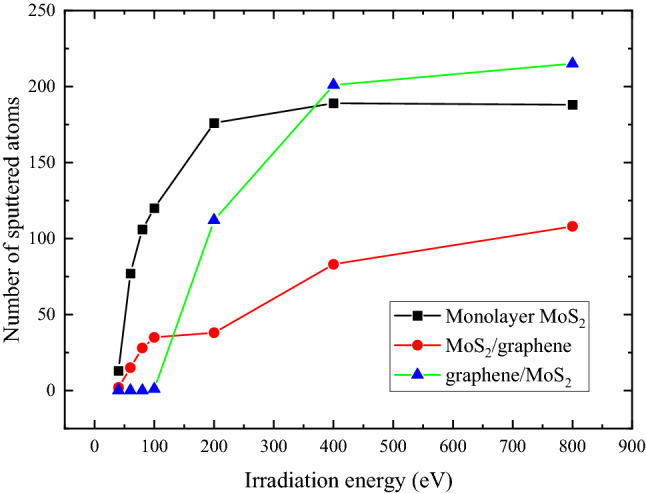
Dependence of the number of sputtered atoms in MoS_2_ layer on irradiation energy for three study cases. The irradiation dose is 6.6 × 10^13^ /cm^2^.

Figure 5 gives the variation of the number of sputtered atoms (atoms being knocked out of the 2D plane) in MoS_2_ layer under different irradiation energies for the aforementioned three cases. It clearly shows that the number of sputtered atoms in MoS_2_ for MoS_2_/graphene is always lower than monolayer MoS_2_ case, demonstrating a perpetual beneficial effect of the bottom-layer graphene regarding the irradiation damage on MoS_2_. For graphene/MoS_2_, there is no damage on MoS_2_ if the irradiation energy is smaller than 100 eV, then the number of sputtered atoms quickly climbs up to surpass the results in MoS_2_/graphene case. It even becomes the most severely damaged when the irradiation energy is above 400 eV, indicating that the indirect collision dominates under this energy level.

**Figure 6:**
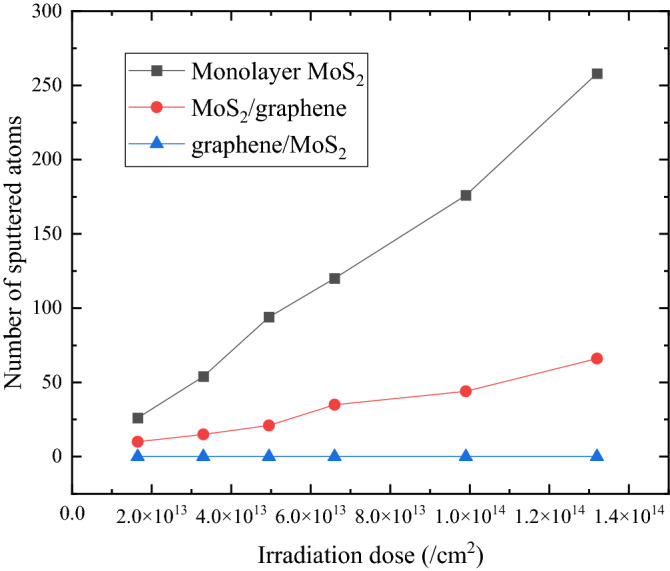
Dependence of the number of sputtered atoms on irradiation dose for three study cases. The irradiation energy is 100 eV.

For the influence of the irradiation dose, we choose the irradiation energy of 100 eV and compare the data of three cases in Fig. 6. It shows that for both monolayer MoS_2_ and MoS_2_/graphene heterostructure, the number of sputtered atoms in MoS_2_ increases almost linearly with the increase of ion dose within the studied range. Due to the protective effect of the bottom graphene, the increase rate of MoS_2_/graphene is smaller. It is expected that at a higher dose beyond the studied range, the number of sputtered atoms would gradually achieve a steady value. In addition, due to the shielding effect of the top-layer graphene, the number of sputtered atoms in graphene/MoS_2_ is always zero under this irradiation energy.

**Figure 7:**
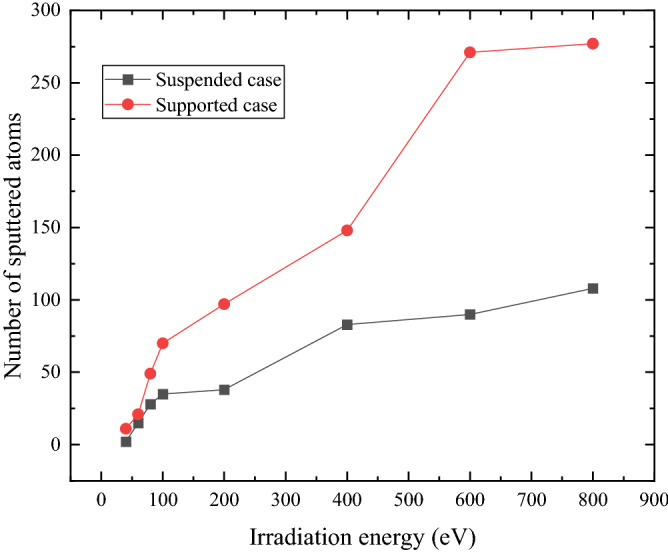
Variation of the number of sputtered atoms in MoS_2_ of MoS_2_/graphene heterostructure for suspended case and supported case. The irradiation dose is 6.6 × 10^13^ /cm^2^.

The actual application of 2D heterostructures normally cannot be separated from the substrate. The existence of substrate has demonstrated to greatly affect the growth and properties of 2D heterostructures^48,49^. To make clear the influence of substrate on the ion irradiation effects of the heterostructures, we plot the relationship between the number of sputtered atoms and irradiation energy for the supported and suspended cases in Fig. 7. It depicts that the number of sputtered atoms for supported case is always larger than suspended case, especially for the high energy condition, the sputtered atoms for supported case increase quickly.

**Figure 8:**
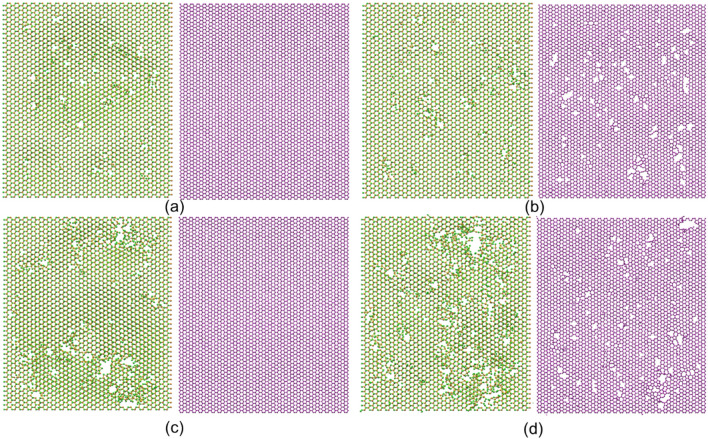
Morphologies of the MoS_2_ and graphene in heterostructures for the suspended and supported cases. (**a**) Suspended case irradiated with 80 eV ions. (**b**) Suspended case irradiated with 400 eV ions. (**c**) Supported case irradiated with 80 eV ions. (**d**) Supported case irradiated with 400 eV ions.

To uncover the mechanism underlying the phenomenon in Fig. 7, we give the configurations of the heterostructures for suspended and supported cases in Fig. 8. It shows that under low ion energy irradiation (80 eV), even though the top-layer MoS_2_ is more severe damaged under substrate supported case, the bottom-layer graphene remains undamaged, which means that the substrate influences the interaction between graphene and MoS_2_. It was described previously that the graphene-MoS_2_ interaction can bring beneficial effect to the irradiation resistance of MoS_2_, therefore, the existence of substrate weakens this beneficial effect. For the high ion energy irradiation (400 eV), the top-layer MoS_2_ is more severe damaged under supported case, in addition, the bottom-layer graphene is also a little bit more damaged under supported case. Especially, there exists some defects in the un-irradiated sites for the supported case. Under high energy irradiation, the impact of ions not only induced the direct collision with the heterostructure layers, but also it resulted in the sputtered of substrate atoms, which could generate second collision (indirect collision) between the substrate atoms and heterostructure atoms. The indirect collision from the substrate can intensify the damage effect in MoS_2_, and due to the random sputtering of the substrate atoms, there would be some defects randomly generated in graphene. Besides, the existence of substrate can also lead to the rebound of the carbon atoms in graphene, which would generate the indirect collision with the atoms in MoS_2_ as well. Therefore, there exists two different mechanisms for the ion irradiation phenomena under the supported case, i.e., the weakened MoS_2_-graphene interaction, and the generation of indirect collision due to the existence of substrate. Under low irradiation energy, only the weakened MoS_2_-graphene interaction mechanism exists, with the increase of irradiation energy, the generation of indirect collision mechanism dominates eventually.

### Uniaxial tensile properties

2D materials and the heterostructures exhibit special mechanical behaviors such as high in-plane strength and stiffness, banding flexibility, which are highly valuable for their applications^50^. The ion irradiation process could provide tailored mechanical behavior to heterostructures if the generation of defects could be controlled. To understand the influence of the ion irradiation on the mechanical properties of the MoS_2_/graphene heterostructure, we conduct uniaxial tensile test simulations for the ion irradiated heterostructures with different irradiation parameters.

**Figure 9:**
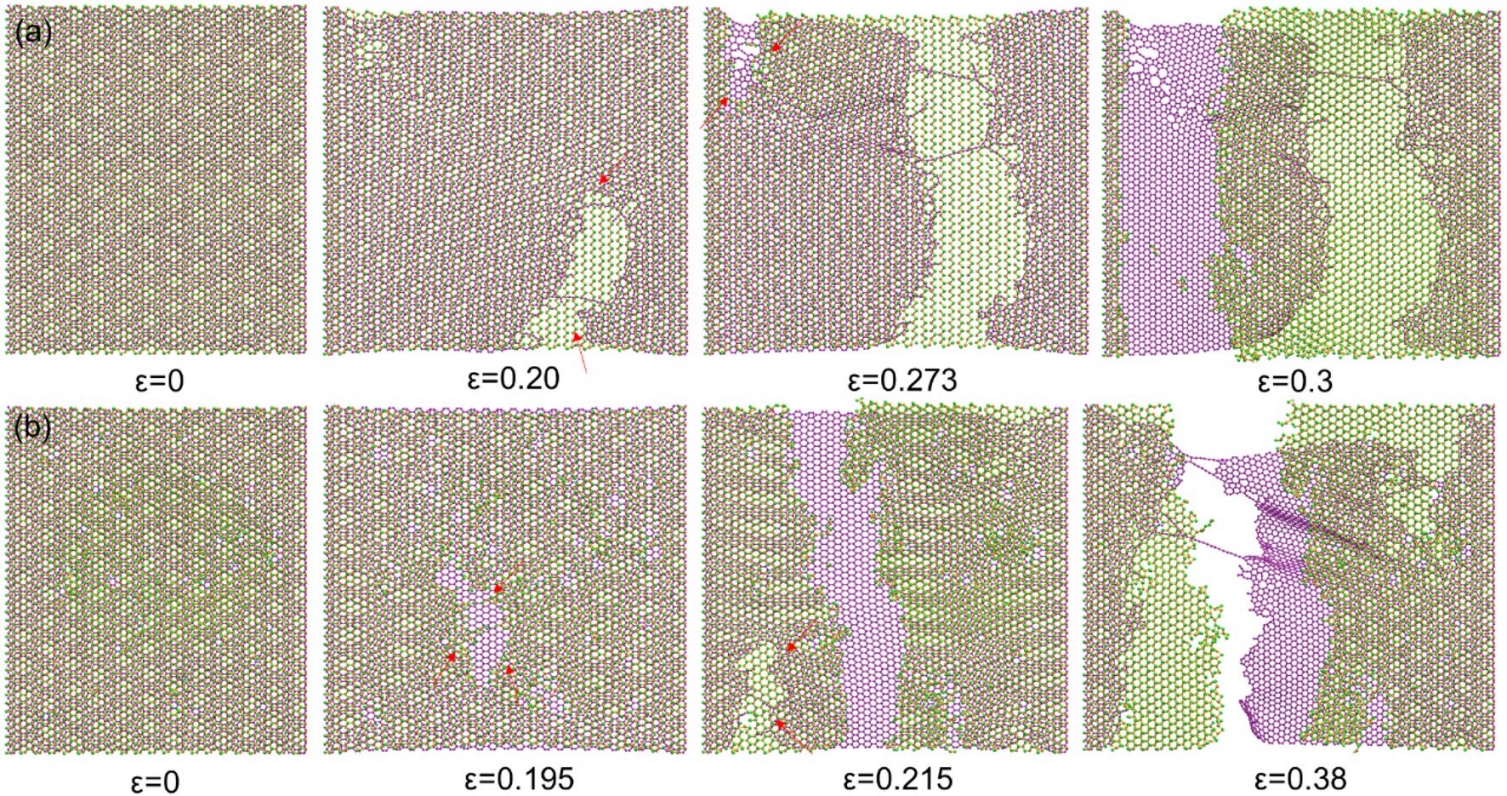
Typical configurations of the MoS_2_/graphene heterostructure under different tensile strains. (**a**) Original MoS_2_/graphene, (**b**) irradiated MoS_2_/graphene. The red arrows are used to mark the initiation sites of the cracks. The ion irradiation energy is 100 eV and irradiation dose is 6.6 × 10^13^ /cm^2^.

The typical configurations of the heterostructure under different tensile strains are provided in Fig. 9 (dynamic stretching processes are given in the supplementary materials S1 and S2), which indicates that both original MoS_2_/graphene and ion irradiated MoS_2_/graphene exhibit a two-stage fracture process under tensile loading. The phenomenon of a two-stage process is attributed to the different tensile behaviors of MoS_2_ and graphene layers. However, a quite different tensile fracture behavior could be observed. Firstly, for the original MoS_2_/graphene heterostructure, the initiation of cracks for the first and second stages happens at a larger strain when compared to the ion irradiation case (0.2, 0.273 vs. 0.195, 0.215), indicating a reduced fracture strain due to the ion irradiation process. Secondly, the cracks of the original MoS_2_/graphene heterostructure initiate at the bottom-layer graphene (first stage), then the cracks start to initiate at the top-layer MoS_2_ at a larger strain (second stage). While for irradiated MoS_2_/graphene heterostructure, the fracture sequence is reversed, i.e., the initiation of crack for the first stage happens at top-layer MoS_2_, and the initiation of crack for the second stage happens at bottom-layer graphene. The reduced fracture strain in irradiated MoS_2_/graphene is owing to the generated defects, which is commonly observed in the irradiated 2D materials. For the reversed fracture sequence, it is also related to the induced defects. The pristine perfect MoS_2_ layer has a larger fracture stress/strain compared to the perfect graphene layer, suggesting a preferential fracture process in graphene, as observed in Fig. 9a. While for the MoS_2_/graphene under 100 eV ion irradiation, the defects are mainly generated in top-layer MoS_2_, resulting an inferior mechanical tensile strength compared to graphene. Therefore, the tensile stretching process leads to the cracks starting from the damaged MoS_2_.

**Figure 10:**
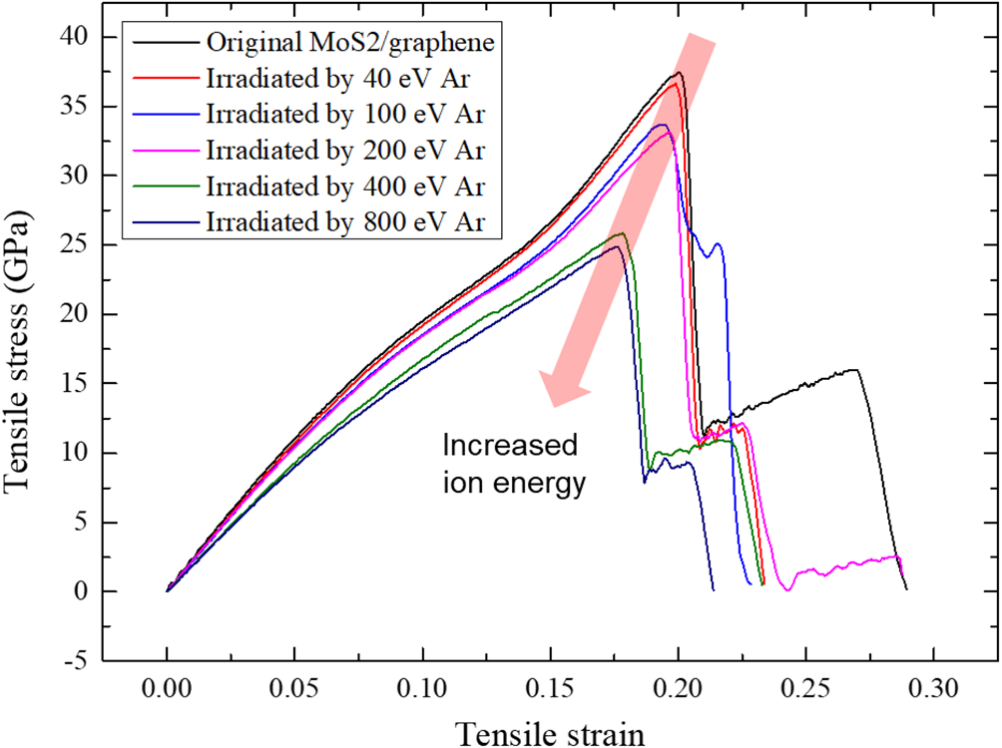
Tensile stress–strain curves of the MoS_2_/graphene heterostructure irradiated with different energies. Note, the stress–strain data was extracted every 500 timesteps.

Figure 10 gives the tensile stress–strain curves of the irradiated MoS_2_/graphene heterostructure at different irradiation energies. It clearly shows that all the structures behave a two-stage fracture process, owing to the different tensile behaviors of MoS_2_ and graphene layers, and the ion irradiation induced defects. The second fracture stage represents the continuous stretching process of top-layer MoS_2_ or bottom-layer graphene, depending on the ion irradiation parameters. The original MoS_2_/graphene has longest second fracture stage due to the defect-free feature, while the irradiated heterostructures have different lengths of second stage due to the distinct defects induced under the different situations. Besides, it is seen that the fracture stress (maximum stress) decreases with the increase of irradiation energy, which corresponds to the increased sputtered atoms with the increase of ion energy. The Young’s modulus also decreases with the increase of ion energy.

### Reduction of the interlayer distance

The experimentally synthesized 2D heterostructures usually exhibit weak interlayer coupling due to the loose interaction induced by the large interlayer distance. Ion irradiation could induce impact force to the 2D layers, bringing defects into the 2D crystal and meanwhile, induce the modulation of the interlayer distance.

**Figure 11:**
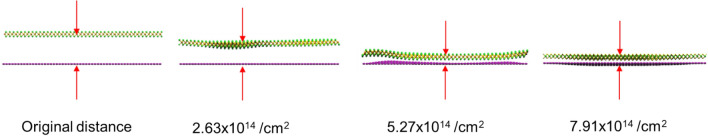
Reduction of interlayer distance in MoS_2_/graphene heterostructure owing to ion irradiation. The ion irradiation energy is 10 eV.

**Figure 12:**
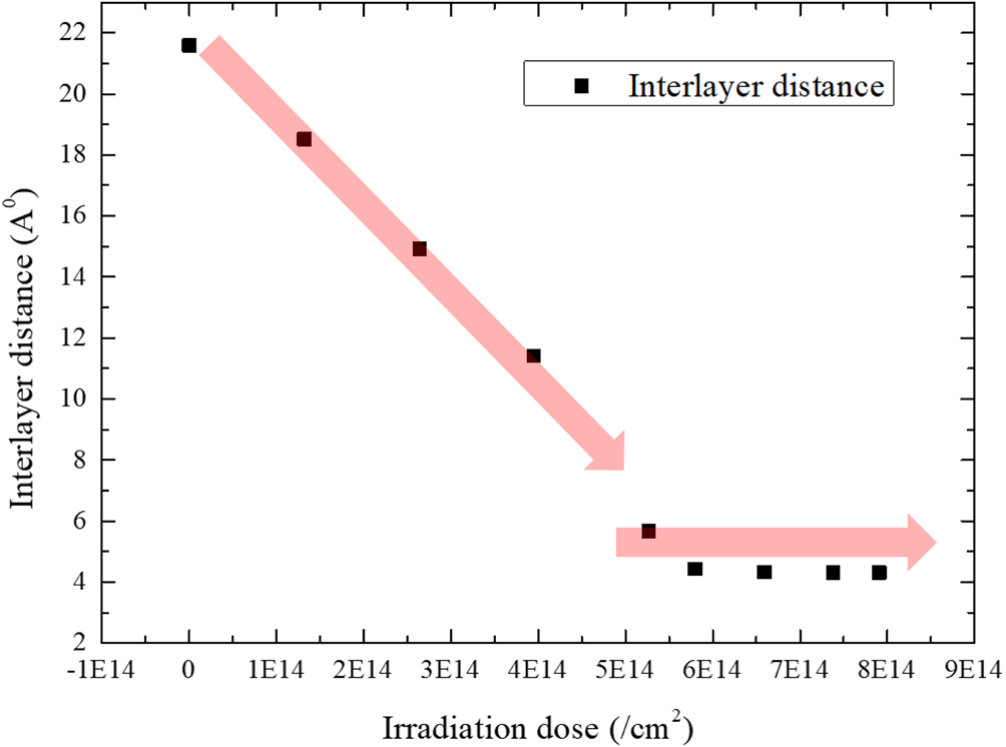
Variation of the interlayer distance in MoS_2_/graphene heterostructure under ion irradiation with different doses. The irradiation energy is 10 eV.

Figure 11 gives the variation of the interlayer distance of the heterostructure at a low energy (10 eV) ion irradiation, for which the initial interlayer distance is set as 2 nm, and the edges of the bottom-layer graphene is fixed. The small dose of ions (2.63 × 10^14^/cm^2^) could induce a slight of the distance due to the limited impact momentum, while at a large ion dose (7.91 × 10^14^/cm^2^), the interlayer distance achieves a much smaller value. Figure 12 plots the dependence of the interlayer distance with the ion irradiation dose. It demonstrates that the distance would decrease almost linearly with the increase of ion dose at first, then it gradually achieves a stable value after the ion dose is large enough. The equilibrium interlayer distance is around 4.3 Å, which is close to the distance with the minimized potential energy. It was already experimentally demonstrated that the ion beam irradiation could be used to adjust the interlayer distance of the 2D heterostructures, further resulting in the control of optical properties^27^. The precise modulation of the interlayer distance is significant to other properties as well, which would be the focus of our future studies.

**Figure 13:**
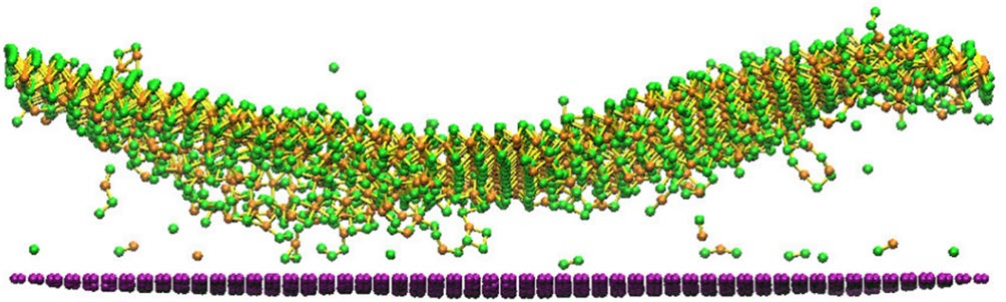
Morphologies of the MoS_2_/graphene heterostructures irradiated with 80 eV, 5.27 × 10^14^/cm^2^ ions.

However, the impact force would also bring defects to the 2D crystals if the irradiation energy is high. As shown in Fig. 13, lots of sputtered atoms and generated defects would exist at a higher ion energy, which indicates that in actual application, the vacancy defects may coexist with the phenomenon of reduced interlayer distance. As a result, the irradiation energy must be precisely controlled to alleviate the side effect of the defects. Meanwhile, the study of the performance of 2D heterostructure with the coexistence of reduced interlayer distance and induced defects is interesting and should receive more attention.

## Conclusion and prospective

Based on classical molecular dynamics simulations, this paper studied the ion irradiation induced phenomena in 2D MoS_2_/graphene heterostructure. The main conclusions are as follows.

1. Under ion irradiation, there would be some vacancies generated in the 2D crystals, while the 2D layers response differently to ion irradiation. For the low energy ion irradiation, the top-layer 2D crystal could shield the bottom-layer, while for the high energy ion irradiation, the indirect collision induced by the top-layer would intensify the irradiation-induced damage into the bottom layer. The bottom-layer 2D crystal would always relieve the defect generation in top layer, regardless of the ion irradiation energy. The irradiation induced vacancies would increase linearly with the increase of ion dose for different study cases. And the substrate would reinforce the irradiation effect because of two mechanisms, i.e., the weakened interlayer coupling in heterostructure at low ion energy and the indirect collision at high ion energy.
2. Ion irradiation would result in the reduction of tensile fracture stress and strain, and the reversed tensile fracture sequence for MoS_2_/graphene heterostructure due to the generated defects. The tensile behavior is highly dependent on the irradiation parameters.
3. The interlayer distance in 2D MoS_2_/graphene heterostructure could be well controlled at a certain ion irradiation dose and energy. While relatively high ion irradiation energy would lead to the coexistence of the defects with reduced interlayer distance.

This study is meaningful for the application of ion irradiation on nanomanufacturing. The future research would focus on the electronic performance modulation and the actual application of ion beam irradiation on tuning the performance of 2D heterostructure-based devices.

## Supplementary Information

Supplementary Video 1.

Supplementary Video 2.

## Data Availability

The data that support the findings of this study are available from the corresponding authors upon reasonable request.
